# Evaluation of the effect of delivery type on dynamic thiol-disulfide balance: a prospective randomized controlled study

**DOI:** 10.1590/1806-9282.20240295

**Published:** 2024-12-02

**Authors:** Merve Keskin Paker, Mehmet Zahit Çıracı, Hayrullah Yazar, Hilal Uslu Yuvaci

**Affiliations:** 1 Sakarya University, Department of Obstetrics and Gynecology – Sakarya, Turkey.; 2 Sakarya University, Department of Medical Biochemistry – Sakarya, Turkey.

**Keywords:** Delivery, Cesarean, Vaginal birth, Oxidative stress, Thiol-disulfide

## Abstract

**OBJECTIVE:**

Thiols play an essential role in eliminating oxidative stress in cells. We aimed to evaluate the oxidative stress occurring during labor with thiol-disulfide balance.

**METHODS:**

A total of 97 healthy pregnant women in the 37th–41st gestational weeks who underwent delivery between June 2021 and August 2022 were included in the study. Elective cesarean section group 1 (n=33), vaginal deliveries group 2 (n=29), and cesarean section due to cephalopelvic disproportion group 3 (n=35) were determined. Blood samples were taken before delivery, and from the umbilical artery just after the fetus and placenta were delivered. Serum native thiol (μmol/L), total thiol (μmol/L), and disulfide (μmol/L) were measured. Disulfide/native thiol (%), disulfide/total thiol (%), and native thiol/total thiol (%) ratios were calculated.

**RESULTS:**

No significant difference was observed in thiol values in maternal blood (p>0.05). There was a significant difference between the groups in terms of native/total thiol (p=0.017), disulfide/total thiol (%) (p=0.018), and disulfide/native thiol (%) (p=0.018) in cord blood.

**CONCLUSION:**

In the literature, evidence concerning the impact of the mode of delivery on the level of neonatal and maternal oxidative stress is inconsistent. It is not clear whether parturition accompanied by pain, as in spontaneous vaginal delivery, poses more oxidative stress than a planned operative delivery. Prospective studies in larger cohorts are needed to better understand the impact of oxidative stress on pregnant women and neonates.

## INTRODUCTION

There has been a recent increase in cesarean section (CS) rates compared to the 15% recommended by the World Health Organization, and the worldwide rate of CS has quadrupled in the last two decades. The CS rate in Turkey is 52.4%, according to the latest data^1^. Many studies on long-term outcomes have revealed that infants delivered via CS have an increased risk of type 1 diabetes mellitus, asthma, allergies, gastrointestinal disorders, childhood leukemias, and testicular cancer^2^. The underlying mechanisms related to these outcomes are uncertain.

Perinatal stress during childbirth leads to permanent changes in neuroendocrine and behavioral responses in the newborn. The stress during labor is essential for preparation for extrauterine life and survival in the transition from the fetal to the neonatal period. With the sympathoadrenal activation it induces, this stress facilitates the generation of the fuel required for the hypoxic journey through the birth canal and adaptation to a dry environment involving respiration through the lungs by transitioning from an aqueous environment where the exchange of oxygen occurs via the placenta. This way, it makes breathing air easier right after birth. This stress also triggers the preparation of the central nervous and inflammatory defense systems for optimal life outside the uterus. Thus, in the infant, it causes an increase in stress hormones, including catecholamines and cortisol. These high secretion levels of stress hormones also trigger hormones and cytokines that participate in inflammatory defense pathways. In this process, the stress experienced during birth by infants varies depending on their mode of delivery^3^.

Oxytocin affects central and peripheral nervous systems, social and sexual behaviors, and motherhood^4^. In recent years, it has been revealed that oxytocin could trigger wound healing and take part in the immune system and inflammatory processes. These effects were explained by the presence of oxytocin receptors in the thymus, and it was proven that these receptors act as acute-phase reactants and interleukins^5^. Hence, oxytocin improves the organism's response to stress with its anxiolytic and antioxidant effects^6^.

For this reason, infants born via CS before the onset of labor and the secretion of oxytocin display a lack of fluctuations in catecholamine levels compared to those born via vaginal delivery after oxytocin secretion. This stress occurs faster in infants with CS than vaginal delivery. CS can cause this maladaptive situation and has been associated with increased neonatal morbidity in the short term^2^.

Thiol-disulfide homeostasis stabilizes protein structures and regulates protein functions, receptors, carriers, Na-K channels, and transcription. It also functions critically in antioxidant defense, detoxification, apoptosis, enzyme activity regulation, transcription, and cellular signal transduction mechanisms. Abnormal thiol/disulfide balance levels are found in the pathogenesis of various diseases. Therefore, identifying dynamic thiol-disulfide homeostasis can provide valuable information regarding various normal or abnormal biochemical processes^7^.

**Figure 1:**
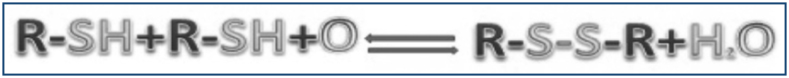
Thiol-disulfide homeostasis^8^.

During pregnancy, the oxygen requirement of tissues increases (Figure 1). The increase in oxygen concentrations leads to oxidative stress by increasing the production of free radicals. It has been demonstrated that oxidative stress levels are significantly higher in women who give birth vaginally compared to CS. Disulfide levels, which reflect oxidative stress, were reported to be higher in the vaginal delivery group compared to the CS group. In contrast, native thiol levels, which reflect antioxidation, were also greater in the vaginal delivery group^9^.

Oxidative stress increases during labor, and this results in the activation of antioxidant mechanisms. This study aimed to investigate the effects of stress during labor on oxidative damage and fetal outcomes. For this purpose, changes in the thiol-disulfide balance between groups were investigated to assess oxidative damage.

## METHODS

This study was planned with a prospective case-control design. All patients signed consent forms after being informed about the study protocol. The study was approved by the Sakarya University Ethics Committee (project ID: E-16214662-050.01.04-34514-123) and was implemented as per the principles of the Declaration of Helsinki.

### Population

Women who gave birth in the Obstetrics and Gynecology Department of the Sakarya Research and Training Hospital between June 2021 and August 2022 were included in the study. Age >40 years, multiple pregnancies, maternal type 1 or 2 diabetes mellitus, gestational diabetes, chronic hypertension, preeclampsia, connective tissue diseases, chronic kidney or liver diseases, or hematological diseases were excluded. Preterm delivery (gestational week <37), small-for-gestational-age infants (birth weight <2 SD), neonatal asphyxia (Apgar score <7, first and fifth minute), chromosomal diseases, or congenital infections were excluded. During follow-up, 18 mother–baby pairs were excluded from the study because ten patients required emergency CS, three patients had increased blood pressure, and five newborns had small for gestational age (SGA). Thus, a total of 97 pregnant women were included in the study. Their age, education level, gestational week, gravidity, parity, height, weight data, newborn birth weight, gender, and Apgar scores were recorded.

The patients with a history of CS were considered the elective CS group (Group 1), spontaneous vaginal deliveries were considered the VD group (Group 2), and CS due to cephalopelvic disproportion observed during their normal labor process, the malposition of the fetal head, or insufficient uterine pushing forces were considered the dystocia group (Group 3).

### Blood sample collection and analysis

Cord blood samples were collected from the maternal side after clamping and cutting the cord. Serum samples were collected in yellow-capped tubes with a gel activator (Becton Dickinson and Company, New Jersey, USA). After keeping them for 30 min, the samples were centrifuged in cooled devices for 10 min. Until the day of the analyses, the samples were kept at −80°C at the biochemistry laboratory of the Sakarya Research and Training Hospital. Total thiol and native thiol levels were determined using the method developed by Erel and Neşelioğlu^10^. The measurements were made using Rel Assay Diagnostics branded kits and an autoanalyzer (Beckman Coulter, AU 680, serial number: 2016024580, Tokyo, Japan). To evaluate thiol-disulfide homeostasis, total thiol levels, native thiol levels, disulfide levels, and the ratios of native thiol/total thiol, disulfide/total thiol, and disulfide/native thiol were used. Disulfide levels were calculated using total and native thiol levels (μmol/L). The ratios were determined using total thiol, native thiol, and disulfide values.

### Statistical analyses

The statistical analyses of the data were carried out with SPSS (Statistical Package for the Social Sciences; SPSS Inc., Chicago, IL) 22 package program. The descriptive data are presented with "n" and % values for the categorical variables and standard deviation (X±SD) values for the continuous variables. Chi-squared analysis (Pearson chi-squared) was used to compare the categorical variables. The normality of the distribution of the continuous data was tested using the Shapiro-Wilk test. A one-way ANOVA was used for the normally distributed data to compare variables among more than two groups. In contrast, the Kruskal-Wallis test was used for the non-normally distributed data. In the analyses, the level of statistical significance was accepted as p<0.05.

## RESULTS

The sample of the study included 97 women and their newborns: Group 1 (n=33), Group 2 (n=29), and Group 3 (n=35). A statistically significant difference was found among the groups in terms of age (p=0.012), while this difference was only between Groups 1 and 3, where Group 1 had a significantly higher mean age (Table 1).

**Table 1:** Maternal characteristics compared across groups.

		Group 1	Group 2	Group 3	p
		X±SD	X±SD	X±SD	
Age		29.5±4.5^a^	26.6±6.1a,b	26.3±5.4^b^	0.012*
Height		162.8±4.4	163.2±4.0	164.1±5.9	0.494**
Weight		69.4±11.2	71.0±9.0	74.3±11.9	0.168**
BMI		26.3±4.8	26.6±2.9	27.6±4.4	0.406**
Gestational week		38.7±0.6	38.8±2.0	39.1±1.4	0.088*
Occupation, n (%)	Homemaker	28 (84.8)	26 (89.7)	25 (71.4)	0.144***
	Working	5 (15.2)	3 (10.3)	10 (28.6)	
Smoker, n (%)	Yes	8 (24.2)	3 (10.3)	6 (17.1)	0.356***
	No	25 (75.8)	26 (89.7)	29 (82.9)	
Consumes alcohol, n (%)	Yes	1 (3.0)	0 (0.0)	0 (0.0)	0.639***
	No	32 (97.0)	29 (100.0)	35 (100.0)	
Exercises, n (%)	Yes	1 (3.0)	1 (3.4)	0 (0.0)	0.534***
	No	32 (97.0)	28 (96.6)	35 (100.0)	
	No	1 (3.0)	28 (96.6)	32 (91.4)	

* Kruskal-Wallis analysis,

** One-way ANOVA,

*** Chi-squared test.

a,b Source of significant difference. SD: standard deviation; BMI: body mass index. Statistically significant values are indicated in bold.

There was no statistically significant difference among the groups in terms of birth weight (p=0.089), first-minute Apgar score (p=0.336), fifth-minute Apgar score (p=0.337), and gender (p=0.099) of the infants.

There was no significant difference among the groups in terms of their maternal blood thiol values (p>0.05).

There were significant differences among the groups in terms of their cord blood native/total thiol (p=0.017), disulfide/total Thiol (%) (p=0.018), and disulfide/native thiol (%) (p=0.018) values, and these differences were significant for all three values only between Groups 1 and 2, where Group 1 had higher native/total thiol ratios and lower disulfide/total thiol and disulfide/native thiol ratios (Table 2).

**Table 2:** Cord blood thiol levels compared across groups.

	Group 1	Group 2	Group 3	p*
	X±SD	X±SD	X±SD	
Total thiol	6561.8±1989.3	6137.4±1445.3	7003.6±2119.9	0.337
Native thiol	4645.6±1580.6	4133.3±1115.9	4761.0±1398.3	0.182**
Disulfide thiol	958.1±249.2	1002.1±208.0	1121.3±484.9	0.471
Reduced thiol ratio Native/total thiol	70.0±4.5^a^	66.9±3.6^b^	68.3±5.7a.b	0.017
Oxidized thiol (disulfide) ratio Disulfide/total thiol (%)	15.0±2.2^a^	16.5±1.8^b^	15.8±2.8a.b	0.018
Thiol oxidation reduction ratio Disulfide/native thiol (%)	21.7±4.7^a^	24.9±3.8^b^	23.7±6.9a.b	0.018

* Kruskal-Wallis analysis,

** One-way ANOVA. SD: standard deviation.

a.b The group from which the difference originates. Statistically significant values are indicated in bold.

## DISCUSSION

This study is the first to include a group requiring CS after the onset of active labor, showing the extent to which birth alone affects oxidative stress. In the literature, the evidence on the effects of mode of delivery on neonatal and maternal oxidative stress levels must be more consistent. It is unclear whether childbirth accompanied by pain, as in spontaneous vaginal labor, creates more oxidative stress than a planned operative childbirth process. Previous studies have investigated modes of labor and their effects on newborns based on different oxidative stress markers^11-13^.

In our study, thiol balance levels in maternal and fetal cord blood samples were measured, and the thiol-disulfide balance in newborns is affected by their mode of delivery. Despite the increases in thiol-disulfide levels in the spontaneous labor and emergency CS groups, there was no significant difference among the groups regarding their neonatal Apgar scores.

In a study, total antioxidant status, erythrocyte glutathione peroxidase (GPx) activity, and erythrocyte superoxide dismutase (SOD) activity parameters were compared to the women who underwent elective CS; those who gave vaginal birth showed elevated placental oxidative stress markers^11^.

In another study, GPx activity, SOD activity, and uric acid levels were noticeably higher in the elective cesarean section (ECS) group than in the VD group. As a result, a significant relationship was shown between delivery modes and oxidative stress markers^12^.

In a systematic review, no significant difference was found in the neonatal oxidative stress measurements of the elective CS and uncomplicated vaginal labor groups. The authors explain the diversity of results by different markers used to measure oxidative stress and different measurement methods^13^.

In the literature so far, the investigation of the effects of modes of delivery on maternal and neonatal dynamic thiol-disulfide homeostasis is low. Considering the low number of such studies, our study, which used this new analytical approach, will contribute to the relevant literature.

Ulubas Isik et al. showed that thiol-disulfide homeostasis did not differ between the VD and CS groups before childbirth. As opposed to the case in our study and other studies in the literature, they found disulfide levels to be lower in infants born via CS than those born vaginally^14^.

In another study, two groups were compared based on their delivery modes. The CS group had significantly lower native thiol, total thiol, disulfide, and disulfide/total thiol ratio values than the VD group. Consequently, again, like in our study, the level of oxidative stress in vaginal childbirth was higher than that in CS^10^. ECS and emergency CS (EmCS) groups were compared in a different study by the same authors. The ECS group had significantly lower native thiol, total thiol, disulfide, and disulfide/total thiol ratio values than the EmCS group. Although oxidative stress levels increased in the EmCS group, as in our study, the groups did not differ significantly in terms of their neonatal Apgar scores^15^.

Furthermore, the third group in our study, which included patients who started vaginal labor but needed CS afterward, was a specialized group different from those in the relevant literature.

Another study did not observe a significant difference in maternal and cord blood thiol/disulfide homeostasis parameters between the labor with oxytocin and labor spontaneously progressed groups^16^. Likewise, Erdal et al. concluded that dynamic thiol-disulfide homeostasis could contribute to monitoring oxidative stress in the infants of patients whose labor is induced using oxytocin and screening diseases that can develop in connection with oxidative stress^17^.

Our results revealed that different modes of delivery affected dynamic thiol-disulfide homeostasis. The oxidative stress levels of both the vaginal labor group and the dystocia group were determined to be higher than the levels of the elective CS group. While previous studies have compared VD and ECS groups, a group that needed to undergo CS after the onset of active labor has not been examined. This showed us the extent to which labor affected oxidative stress by itself. This study will contribute substantially to the literature because it is the only study to include such a group. However, prospective studies of broader cohorts are needed to understand better the effects of oxidative stress on pregnant women and newborns.

## LIMITATIONS

This study had some limitations. First, our ECS group age was unexpectedly higher than the mean ages of the other two groups. The second limitation of the study was the absence of a long-term follow-up period to changes in thiol/disulfide homeostasis in the postpartum period. Finally, the number of patients was small. More prospective randomized controlled studies are needed to confirm our results.

## WHAT DOES THIS STUDY ADD TO THE CLINICAL WORK?

This study showed how much labor affected oxidative stress by itself. It will contribute substantially to the literature because it is the only study to include such a group.

## ETHICS APPROVAL

The study protocol, which was approved by the institutional ethics committee of our university (project ID: E-16214662-050.01.04-34514-123), was implemented per the principles of the Declaration of Helsinki.

## CONSENT TO PARTICIPATE

All patients signed consent forms after being informed about the study protocol.
